# Conversational assessment of cognitive dysfunction among residents living in long-term care facilities

**DOI:** 10.1017/S1041610217001740

**Published:** 2017-09-21

**Authors:** Hikaru Oba, Shinichi Sato, Hiroaki Kazui, Yoshiko Nitta, Tatsuya Nashitani, Akio Kamiyama

**Affiliations:** 1 Department of Psychiatry, Graduate School of Medical Science, Kyoto Prefectural University of Medicine, Kamigyo-ku, Kyoto, Japan; 2 Department of Clinical Thanatology and Geriatric Behavioral Science, Osaka University Graduate School of Human Sciences, Suita-City, Osaka, Japan; 3 Department of Psychiatry, Osaka University Graduate School of Medicine, Suita-City, Osaka, Japan; 4 Baba Memorial Hospital, Sakai-City, Osaka, Japan; 5 Cocolomi Co., Ltd, Shibuya-ku, Tokyo, Japan

**Keywords:** dementia, behavioral and psychological symptoms of dementia (BPSD), nursing homes, quality of life (QoL), cognitive assessment

## Abstract

**Background:**

There are some existing barriers posed by neuropsychological tests that interfere with the assessment of cognitive functioning by staff who work in long-term care facilities. The purpose of this study was to investigate the feasibility of assessing cognitive function through conversation.

**Methods:**

A total of 100 care staff was randomly selected as participants. Each staff member evaluated cognitive function in one to three residents using the Conversational Assessment of Neurocognitive Dysfunction (CANDy), which is a screening test for dementia using conversation. Other scales used were the Mini-Mental State Examination (MMSE), Behavioral Pathology in Alzheimer’ s Disease (BEHAVE-AD), and quality-of-life questionnaire for the elderly with dementia (QOL-D).

**Results:**

A total of 80 care staff members and 158 residents were analyzed. When the CANDy involved an evaluation based on face-to-face communication, it demonstrated significant correlations with the MMSE, BEHAVE-AD, and several indices of the QOL-D (e.g. negative affect and actions, communication ability, restless, and spontaneity and activity). In contrast, when the CANDy involved an evaluation based on an impression of a typical conversation, it only demonstrated significant relationships with the MMSE and the spontaneity and activity index of the QOL-D.

**Conclusions:**

Conversational assessment is a useful means to assess cognitive functioning and to promote interactions between residents and care staff in long-term care facilities.

## Introduction

Cognitive dysfunction, which includes deficits in memory, orientation, language, and decision-making, is exhibited by individuals with dementia. Moreover, there are also several behavioral and psychological symptoms of dementia (BPSD), which involve wandering, violence, hallucinations, and delusions. Therefore, when caring for individuals with dementia, it is important to assess cognitive functioning because declines in cognitive functioning are related to increases in BPSD (Cohen-Mansfield and Libin, 2005; Starr and Lonie, 2007; Zahodne *et al*., 2015).

However, there are some existing barriers that make it difficult for the staff who work in long-term care facilities to assess the cognitive functioning of residents using neuropsychological tests. First, care staff fear that performing these neuropsychological evaluations will damage their relationship with the residents. Many neuropsychological tests are invasive because in order to measure one's ability, answers to test questions inherently have correct or incorrect answers. In some cases, this has caused both the test subject and the examiner to feel distress following the administration of the neuropsychological test (Tiberti *et al*., 1998; Cahill *et al*., 2008; Lai *et al*., 2008). This resulting interpersonal distress may affect the quality of patient care because the patient–clinician relationship has an impact on healthcare outcomes (Kelley *et al*., 2014). Second, staff lack the necessary time to conduct neuropsychological tests, as they spend 60% of their working time assisting with personal care, such as bathing, dressing, and assisting with eating (Mallidou *et al*., 2013). Taken together, staff's interpersonal distress and lack of time make it difficult for them to conduct neuropsychological tests.

In addition, care staff rarely interact with residents, as less than 1% of their working time is spent socializing with residents (Mallidou *et al*., 2013), and most residents spend their days alone (Schreiner *et al*., 2005). Other studies have shown that the interactions between care staff and residents took approximately 2.5% of the workday, and 77% of these interactions were task-oriented, whereas only 15% were social- or relationship-oriented (Ward *et al*., 2008). One reason for the lack of interactions with residents was the healthcare environment's “doing” culture, wherein it is of greater value for care staff to perform tasks in which they appear to be physically occupied (Scott-Findlay and Golden-Biddle, 2005), and interacting with others is not considered a work task. In addition, interaction with residents may often cause physical and emotional strain in the staff because the communication problems of people with dementia are influenced by cognitive and functional impairment, and are also associated with BPSD (Savundranayagam *et al*., 2005). However, the research conducted within long-term care facilities has revealed that having more frequent social interactions is related to positive mood (Beerens *et al*., 2016), and residents desire social interactions (Saunders *et al*., 2012). Therefore, evaluating cognitive function is important to increase interactions between residents and care staff, which may contribute to alleviating the feelings of isolation and loneliness in residents.

Given the above problems noted with conventional neuropsychological tests, recent studies have focused on conversational profiles of older adults to detect dementia. For example, individuals with dementia forget the content of questions, are unable to reply to compound questions, display a lack of working memory in their interactions, have difficulties producing words, and have difficulties sustaining the interaction (Rousseaux *et al*., 2010; Elsey *et al*., 2015; Jones *et al*., 2016). Assessing cognitive function based on conversation characteristics can provide useful information in helping diagnose dementia (Jones *et al*., 2016). Moreover, given the difficulties posed by conventional neuropsychological tests and care staff's work environment, the use of a conversational assessment is more advantageous in long-term care facilities because it is less invasive and requires interaction between care staff and residents in order to evaluate conversational profiles. Therefore, conversational assessments by staff could contribute to improving the care practice of long-term care facilities.

One screening test that has been developed in this regard is the Conversational Assessment of Neurocognitive Dysfunction (CANDy), which is designed to detect dementia through conversation (Oba *et al*., 2017). It has been suggested by Oba *et al*. (2017) that not only does the CANDy avoid distress between the examiner and older adults, but it also promotes social interaction. However, the CANDy was previously validated in a sample of physicians and psychologists, who have adequate knowledge about and clinical experience with dementia (Oba *et al*., 2017); therefore, its validity when used by other professionals is unclear. Given this, the purpose of the current study was to investigate the validity of the CANDy for use by care staff as a conversational assessment for cognitive function, as well as to examine the potential for conversation to promote interactions between care staff and residents in long-term care facilities.

## Methods

### Procedure

The study was conducted in ten long-term care facilities in the Kinki region of Japan between July and August 2016. We randomly selected 100 care staff members whose main work involved direct care of the residents. Participants each evaluated between one and three residents. The order of the measures in the questionnaire was as follows: demographic characteristics of the staff and residents, CANDy, Mini-Mental State Examination (MMSE), quality-of-life questionnaire for the elderly with dementia (QOL-D), and Behavioral Pathology in Alzheimer’ s Disease (BEHAVE-AD). All measures were completed by staff simultaneously, after which they submitted the questionnaires to the survey manager in each facility. After all questionnaires had been collected in that facility, the survey manager sent it to the researcher by mail.

This study was approved by the Ethics Committee of the Department of Human Sciences, Osaka University. Information regarding the objective of the survey, benefits, and disadvantages of answering the questionnaires, methods by which to return the questionnaires, and informed consent were written on the face sheet. Facility residents were also provided by staff with a verbal explanation of the survey objectives, and the benefits and disadvantages of participation before beginning the survey. When submitting the questionnaire to the survey manager, staff were asked to insert the questionnaire into an envelope provided to them; this was done to identify respondents who answered two or more questionnaires and to protect their answers from interference by survey managers (e.g. if staff had submitted a blank questionnaire, the survey managers might have forced them to answer it before it was submitted to the researcher). Participants and residents who did not provide their informed consent were excluded from the study.

## Measures

### Sociodemographic data

We collected information regarding participants’ age, gender, licensure status, employment status, career length, and duration at present institution, as well as information regarding residents’ gender, age, and level of care.

### Conversational assessment

Conversational assessment of cognitive function was measured by the CANDy, which is a 15-item test that screens for the presence of dementia through daily communication with older adults (Oba *et al*., 2017). The user manual and accompanying assessment sheets for the CANDy can be downloaded from the following website: http://cocolomi.net/candy/en/. The CANDy evaluates the occurrence frequency of certain conversational characteristics (e.g. repeatedly asking the same question during conversation; understanding whether one's conversation partner is being vague) that occur among people with dementia through a ≥30-minute conversation with them. These conversational characteristics were identified through a pilot survey targeting physicians specializing in psychiatry or neurology, clinical psychologists, and care staff (Oba *et al*., 2017). The frequency of these characteristics is rated on a three-point scale from 0 (not seen at all) to 2 (often seen). Scores range from 0 to 30, with higher scores reflecting more severe cognitive dysfunction.

In a previous study in which the CANDy was used by 13 physicians who specialized in psychiatry or neurology and 10 clinical psychologists to evaluate patients with dementia (*N* = 45) (Oba *et al*., 2017), the measure was moderately correlated with the MMSE (*r* = –0.629, *p* < 0.001). Moreover, using a cut-off score of 5/6, it correctly distinguished between patients with Alzheimer's disease (AD) (*N* = 29) and healthy elderly (*N* = 73) with a sensitivity of 86.2% and a specificity of 94.5%. The CANDy for healthy elderly was evaluated by 13 trained telephone counselors in elderly care services. The Cronbach's *α* of the CANDy was 0.90 according to the physicians and clinical psychologists, and 0.85 according to the telephone counselors. Although these past studies had some limitations, such as a small sample size and not showing a benchmark diagnosis of AD, the CANDy can be considered effective in evaluating conversational profiles.

Moreover, although the CANDy is primarily conducted via face-to-face communication with residents, it can also provide an assessment by having the examiner give his or her impression of a typical conversation with a resident, provided that the examiner is familiar with that resident. Indeed, the correlation between the CANDy and the MMSE when it was used to evaluate residents by giving an impression of a typical conversation was –0.647 (*p* < 0.01). Therefore, participants were asked to clarify whether they evaluated residents via face-to-face communication or by giving their impression of a typical conversation. Participants were also asked the conversation time required to complete the assessment.

### Conventional screening test of dementia

The MMSE (Folstein *et al*., 1975) is screening test for dementia that is used worldwide. This test consists of 11 items that measure orientation to time and place, registration, attention and calculation, recall, naming, repetition, ability to follow a three-stage command, reading, writing, and copying. Scores range from 0 to 30, with lower scores reflecting more severe cognitive dysfunction.

### Psychiatric symptoms

The BEHAVE-AD (Reisberg *et al*., 1987) is a semi-structured interview conducted with caregivers who are familiar with individuals with dementia. The scale consists of 25 items that evaluate the severity of BPSD, such as violence, wandering, hallucinations, delusions, depression, and anxiety. The degree of severity is rated on a four-point scale that ranges from 0 (nothing) to 3 (severe). Higher scores reflect more severe BPSD.

### QOL

The QOL-D (Terada *et al*., 2002) consists of 31 items classified into six indices that are, in turn, divided into two domains. One domain measures positive aspects of QOL (e.g. positive affect, attachment with others, communication ability, and spontaneity and activity), and the second domain measures negative aspects (e.g. negative affect and actions, and restlessness). Items are rated on a four-point scale with responses ranging from 1 (none) to 4 (frequent), as well as not applicable. Higher scores on the positive aspects domain and lower scores on the negative aspects domain reflect better QOL. In this study, items on the negative aspects domain were reverse scored, such that higher scores reflected better QOL.

### Statistical analysis

First, we calculated the descriptive statistics of participants’ and residents’ general characteristics, cognitive functioning, psychiatric symptoms, and QOL. For the MMSE, we removed items with missing data when calculating the mean and standard deviation of the total score. We used the sum of item scores of the BEHAVE-AD. We also used the mean of the items of the QOL-D, although we excluded answers of “not applicable.” The reliability of the CANDy was also calculated using the Cronbach's *α* coefficient. Correlations between CANDy scores and other variables were analyzed using Pearson's correlations with Holm's *post hoc* correction to account for multiple tests. Missing values in each measure were handled via pair-wise case deletion. Data were analyzed using R version 3.3.1 (R core team, 2016).

## Results

### Characteristics of residents

A total of 208 questionnaires were collected. Of these, 36 could not be identified, 13 were blank, and 1 contained an unanswered CANDy. Thus, the data of 158 residents (45 male, 112 female, 1 unknown) with a mean age of 83.8 years (*SD* = 6.8 years) were included. These residents were evaluated by 80 care staff members (43 male and 37 female) with a mean age of 45 years (*SD* = 9.8 years) and an average career length of 12.2 years (*SD* = 7.5 years). The characteristics of participants are shown in Table 1, the descriptive statistics regarding residents are presented in Table 2, and the descriptive statistics for the CANDy are shown in Table 3. The level of care required by 75.3% of residents ranged from 3 to 5, which is indicative of mild to moderate impairment in activities of daily living (ADLs) and instrumental activities of daily living (IADLs). Residents’ mean scores on measures of cognitive functioning, psychiatric symptoms, and QOL are presented in Table 4. Irrespective of dementia diagnosis, residents’ average MMSE score was < 24 points (14.8 ± 7.2 for the diagnosed group and 17.2 ± 6.7 for the undiagnosed group). The CANDy demonstrated high internal consistency in the current sample (Cronbach's *α* = 0.91).

**Table 1.** Participant descriptive statistics

	*n*	%
Gender		
Male	43	53.8
Female	37	46.3
Age^a^ (*N* = 79)	45.0 ± 9.8 years	
License^b^		
Certified care worker	64	80.0
Social worker	9	11.3
Home helper	14	17.5
Career Length^a^ (*N* = 71)	12.2 ± 7.5 years	
Duration at present institution^a^ (*N* = 78)	5.4 ± 5.9 years	
Employment status		
Full-time	71	88.8
Part-time	9	11.2

*Note. N* = 80. Missing values were excluded from calculating the mean and standard deviation.

a *Mean* ± *SD*. ^b^Multiple answers possible.

**Table 2.** Resident descriptive statistics

	*n*	%
Gender		
Male	45	28.7
Female	112	71.3
Missing values	1	
Age^a^ (*N* = 153)	83.8 ± 6.8 years	
Dementia diagnosis		
Yes	93	60.4
AD	59	69.4
DLB	1	1.2
VD	6	7.1
FTD	1	1.2
Unknown	14	16.5
Other	4	4.7
Missing values	8	
No	61	39.6
Missing values	4	
Level of Care		
None	14	9.1
1	6	3.9
2	3	1.9
3	52	33.8
4	35	22.7
5	29	18.8
6	11	7.1
7	4	2.6
Missing values	4	

*Note. N* = 158 Missing value was excluded in calculating mean and standard deviation.

AD = Alzheimer's Disease, DLB = dementia with Lewy bodies, VD = vascular dementia, FTD = frontotemporal dementia.

a *Mean* ± *SD*.

**Table 3.** Descriptive statistics of the CANDy

	*n*	%
Assessment		
Face-to-face	109	73.2
Impression of typical conversation	40	26.8
Missing value	9	
Frequency of conversation with residents		
For the first time	9	5.7
Rarely	43	27.4
Often	105	66.9
Missing value	1	
Conversation time^a^ (*N* = 121)	29.8 ± 13.9 minutes	
Score on the CANDy^a^ (*N* = 158)	12.7 ± 7.3	

*Note*. *N* = 158. Missing values were excluded from calculating the means and standard deviations.

a Mean ± *SD*.

**Table 4.** Descriptive statistics of the MMSE, BEHAVE-AD, and QOL-D

	*n*	*m*	*sd*
MMSE	148	15.9	7.1
BEHAVE-AD	134	4.7	6.0
QOL-D			
Positive affect	156	3.1	0.7
Negative affect and actions	156	3.5	0.7
Communication ability	157	3.3	0.6
Restlessness	157	3.3	0.7
Attachment with others	157	2.6	1.0
Spontaneity and activity	157	2.6	0.8

*Note*. Missing values were excluded from calculating the means and standard deviations.

MMSE = Mini-Mental State Examination, BEHAVE-AD = Behavioral Pathology in Alzheimer's Disease, QOL-D = quality of life questionnaire for the elderly with dementia.

The selection of the CANDy assessment method differed according to the patients’ dementia diagnosis. For residents who were diagnosed with dementia, the participants more frequently used the impression of the typical conversation method than for residents who had not been diagnosed with dementia (diagnosed = 34.1%, undiagnosed = 18.3%, *χ^2^* = 4.39, *p* = 0.036). By contrast, it did not differ according to the staff characteristics or the age, gender, and care level of residents.

### Correlation between CANDy scores and other variables

Correlations between the CANDy, MMSE, BEHAVE-AD, and QOL-D are presented in Table 5. CANDy scores calculated via face-to-face interaction demonstrated significant correlations with the MMSE (*r* = –0.624, *N* = 104, *p* < 0.001), BEHAVE-AD (*r* = 0.375, *N* = 93, *p* = 0.020), QOL-D communication ability (*r* = –0.500, *n* = 109, *p* < 0.001), QOL-D restlessness (*r* = –0.393, *N* = 109, *p* < 0.001), and QOL-D spontaneity and activity (*r* = –0.458, *N* = 109, *p* < 0.001). In contrast, CANDy scores calculated using an impression of a typical conversation only demonstrated significant correlations with the MMSE (*r* = –0.691, *N* = 36, *p* < 0.001) and QOL-D spontaneity and activity (*r* = –0.499, *N* = 39, *p* = 0.012).

**Table 5.** Pearson's correlations between the CANDy and other measures

	CANDy (All)	*n*	CANDy (face-to-face)	*n*	CANDy (impression)	*n*	MMSE	*n*
MMSE	−0.640***	148	−0.624***	104	−0.691***	36		148
BEHAVE-AD	0.379***	134	0.375*	93	0.385	35	−0.187	125
QOL-D								
Positive affect	−0.235**	156	−0.205	108	−0.280	39	0.175	146
Negative affect and actions	−0.278**	156	−0.267	109	−0.347	38	0.307*	146
Communication ability	−0.436***	157	−0.500***	109	−0.296	39	0.517***	147
Restlessness	−0.369***	157	−0.393***	109	−0.375	39	0.232*	147
Attachment with others	−0.119	157	−0.145	109	−0.012	39	0.123	147
Spontaneity and activity	−0.473***	157	−0.458***	109	−0.499*	39	0.507***	147

*Note*. Pair-wised case deletion was used to handle missing values in calculating the correlations.

*Post-hoc* analyses used Holm's procedure. CANDy = Conversational Assessment of Neurocognitive Dysfunction; MMSE = Mini-Mental State Examination; BEHAVE-AD = Behavioral Pathology in Alzheimer’ s Disease; QOL-D = Quality of life questionnaire for the elderly with dementia.

*p < 0.05, **p < 0.01, ***p < 0.001.

Although the MMSE was significantly correlated with several indices of the QOL-D, including negative affect and actions (*r* = 0.307, *N* = 146 *p* = 0.020), communication ability (*r* = 0.517, *N* = 147, *p* < 0.001), restlessness (*r* = 0.232, *N* = 147, *p* = 0.047), and spontaneity and activity (*r* = 0.507, *N* = 147, *p* < 0.001), it did not correlate significantly with the BEHAVE-AD (*r* = –0.187, *N* = 125, *p* = 0.371), unlike the results for the CANDy.

## Discussion

The results indicated that conversational assessments of cognitive function using the CANDy were associated with the conventional cognitive test. Moreover, the CANDy was associated with BPSD and QOL in particular in case of face-to-face evaluation. The present study suggested that conversational assessment by the care staff is useful to assess cognitive and mental functions in long-term care facilities.

In the present study, correlations between the CANDy and MMSE did not differ based on method of evaluation (i.e. face-to-face communication or impression of typical conversation). These findings suggest that the CANDy can provide a valid assessment of cognitive functioning irrespective of the manner in which the measure is administered. Previous studies suggested that neuropsychological tests evaluating cognitive function are invasive, which can cause feelings of distress and consequently affect the relationship between residents and examiners (Tiberti *et al*., 1998; Cahill *et al*., 2008; Lai *et al*., 2008). Moreover, care staff often lack the necessary time to conduct neuropsychological tests (Scott-Findlay and Golden-Biddele, 2005; Mallidou *et al*., 2013). These factors may interfere with the operation of neuropsychological tests in long-term care facilities. The present study indicated that the average time it took to complete the CANDy was about 30 minutes, which is somewhat longer than is necessary for completing conventional screening tests such as the MMSE. However, the CANDy would nevertheless be a useful tool for assessing cognitive functioning in practice because of its lower invasiveness and fewer time constraints, in that it can be completed through face-to-face interaction or the examiner's impression of a typical conversation with the resident. Moreover, the CANDy can be completed repeatedly over a short period because it evaluates conversational profiles and is not subject to a learning effect. Therefore, the use of conversational assessments in long-term care facilities is considered more feasible than the use of neuropsychological tests.

Previous studies have shown a relationship between cognitive functioning and BPSD (Cohen-Mansfield and Libin, 2005; Starr and Lonie, 2007; Zahodne *et al*., 2015). In this study, significant correlations between the CANDy and the BEHAVE-AD were observed when the former was administered via face-to-face interaction. While the correlation of the CANDy based on examiners’ impressions was not significant, the coefficient was relatively similar to that for the face-to-face interaction. These results suggest that this conversational assessment can be used to obtain information on various neuropsychiatric symptoms, possibly because it is less formal than conventional cognitive tests. This also might mean that conversational assessments can enhance clinical assessment if they are used in conjunction with conventional cognitive tests.

The results of the current study also indicated a relationship between the CANDy and the QOL-D. Importantly, when the CANDy was based on face-to-face interactions, the CANDy scores had stronger correlations with residents’ communication ability as measured by the QOL-D than when it was based on impressions of a typical conversation. These results suggest that a conversational assessment that involves face-to-face communication promotes interaction with the residents. In addition, a proxy evaluation of QOL conducted by care staff may affect their own feelings (Robertson *et al*., 2017). As a result, care staff might discover various characteristics about the residents of which they were previously unaware by having engaged them in conversation. Since research has shown that residents desire social interactions (e.g. Saunders *et al*., 2012), conversational assessments are also a useful communication tool that can promote interactions between care staff and residents (Oba *et al*., 2017).

As with any study, there are some limitations to the current study. First, our findings are limited by a small sample size. In addition, most of the residents were evaluated using face-to-face communication, and few residents were evaluated using an impression of a typical conversation. Given this, correlations between the CANDy that involved impressions of a typical conversation and the BEHAVE-AD and QOL-D might lack statistical power. Moreover, the analysis was based on simple correlations and we did not consider the more complex relationships among the variables (e.g. mediators). Second, the present study was not able to analyze the sensitivity and the specificity to the diagnosis of dementia of the CANDy scores between residents who had been diagnosed with dementia and those who had not because all residents showed a decline in cognitive functioning. In fact, residents in long-term care facilities do not always receive a diagnosis by a physician even when they show cognitive impairment. Additionally, the poor cognitive function of the residents in this study suggest the presence of a ceiling effect of the CANDy – in other words, the CANDy might be less sensitive or reliable because the CANDy score would indicate better cognitive function (the score is near zero) among most patients with lower (i.e. mild) cognitive impairment. Moreover, the test–retest and inter-rater reliability could not be confirmed in either this study. It is necessary to investigate the consistency of the evaluation of the CANDy using repeated evaluations by the same examiner and evaluation by different examiners of the same resident. Third, we did not account for possible biases presented by the order of the scales in the questionnaire. The CANDy came before the MMSE in the questionnaire booklet, but it is possible that the CANDy evaluations might differ if the MMSE was administered first. In addition, the evaluation of the CANDy might be influenced by the performance of other measures. Future studies should use the blind design to evaluate the CANDy and other measures more accurately. Moreover, we found that the assessment method for the CANDy differed according to residents’ dementia diagnosis. Residents with severe cognitive decline might have greater impairments in communication or BPSD, making it difficult for care staff to interact with them. Given these limitations, it is necessary to be cautious in our interpretations of the results. Additionally, we suggest the need for further studies that consider different patient characteristics and a wider range of cognitive function levels, and that test the reliability of the CANDy.

## Conclusion

Despite these limitations, the current study determined that the use of conversational assessment is a helpful means by which to evaluate cognitive function in long-term care facilities. Not only does conversational assessment result in less distress than traditional neuropsychological evaluations, it also promotes interactions between residents and care staff. Given the current findings, we believe that the use of conversational assessment in long-term care facilities is more advantageous than is the use of conventional neuropsychological tests.

## Conflicts of interest

None.

## Description of authors’ roles

All authors were involved in the design of the study. H. Oba searched the literature, collected the data, analyzed the data, and wrote the first draft of the paper. S. Sato, H. Kazui, Y. Nitta, T. Nashitani, and A. Kamiyama collected the data and provided intellectual contributions. S. Sato also supervised the project. All authors reviewed drafts of the paper and approved the final version of the paper for publication.
